# Supplementary material for: Group dominance increases territory size and reduces neighbour pressure in wild chimpanzees
Source: R Soc Open Sci. 2020 May 27;7(5):200577. doi: 10.1098/rsos.200577 (PMC7277268; doi:10.1098/rsos.200577)
Supplement: Demographic and Ranging Information, Model Results Lemoine et al. Group dominance increases territory size and reduces neighbour pressure in wild chimpanzees [file rsos200577supp1.docx]

**ROYAL SOCIETY OPEN SCIENCE**

**Group dominance increases territory size and reduces neighbour pressure in wild chimpanzees**

Sylvain Lemoine^1,2,3,4^, Christophe Boesch^1^, Anna Preis^1,2^, Liran Samuni^1,2^, Catherine Crockford^1,2,3^ and Roman M. Wittig^1,2,3^

^1^Department of Primatology, Max Planck Institute for Evolutionary Anthropology, Deutscher Platz 6, 04103 Leipzig, Germany

^2^Taї Chimpanzee Project, Centre Suisse de Recherche Scientifique en Côte d’Ivoire, 01 BP 1303, Yopougon, Abidjan, Ivory Coast

^3^Department of Human Behavior, Ecology and Culture, Max Planck Institute for Evolutionary Anthropology, Deutscher Platz 6, 04103 Leipzig, Germany

**Electronic Supplementary Material**

**Table S1: Summary of demographic variables and spatial parameters for the four studied communities. Demographic numbers were calculated on a monthly basis. Group sizes include all weaned individuals. Number of mature individuals include all adult (> 12yr) and adolescent (> 10yr) males and females.**

|  | **NORTH** | **MIDDLE** | **SOUTH** | **EAST** |
| --- | --- | --- | --- | --- |
| **Period of study** | 1997-2016 | 1999-2004 | 1999-2016 | 2008-2016 |
| **Mean ± SE group size** | 20.87 ± 0.29 | 8.76 ± 0.3 | 37.44 ± 0.64 | 39.68 ± 0.61 |
| **Min. - Max. group size** | 16 - 32 | 5 - 13 | 22 - 55 | 30 – 49 |
| **Mean ± SE**  **number adult males** | 1.83 ± 0.05 | 1.93 ± 0.12 | 5.11 ± 0.09 | 4.39 ± 0.07 |
| **Min. - Max. number adult males** | 0 - 4 | 1 - 3 | 2 - 7 | 3 – 7 |
| **Mean ± SE**  **number adult females** | 7.40 ± 0.10 | 2.6 ± 0.09 | 12.91 ± 0.27 | 13.37 ± 0.25 |
| **Min. - Max. number adult females** | 5 - 12 | 2 - 4 | 6 – 21 | 9 – 18 |
| **Mean ± SE number mature individuals** | 10.95 ± 0.13 | 5.05 ± 0.28 | 21.97 ± 0.27 | 21.38 ± 0.32 |
| **Min. - Max. number mature individuals** | 7 - 16 | 3 - 8 | 14 – 28 | 15 – 29 |
| **Mean ± SE**  **number nulliparous females** | 1.29 ± 0.06 | 0.64 ± 0.09 | 1.70 ± 0.10 | 2.82 ± 0.11 |
| **Min - Max. number nulliparous females** | 0 - 4 | 0 - 2 | 0 - 5 | 0 – 6 |
| **Mean ± SE**  **annual 95% fixed kernel**  **(territory size)** | 12.67 ± 0.47 km^2^ | 11.02 ± 1.98  km^2^ | 23.36 ± 1.24  km^2^ | 30 ± 1.29  km^2^ |
| **Min - Max. annual 95% fixed kernel (territory size)** | 9.25 - 16.24 km^2^ | 6.42 - 19.14 km^2^ | 15.49 - 36.59  km^2^ | 20.60 - 34.45 km^2^ |

**Table S2: Determinants of yearly territory size for a model using the number of adult males (model 1B).**

| **Random effect** | **Terms** | **Variance** | **Estimate** | **SE** | **χ ²** | **95% CI** | **Df** | **P** |
| --- | --- | --- | --- | --- | --- | --- | --- | --- |
| Group | intercept | 13.303 | 3.503 | 4.895 |  | -6.864 ; 13.248 |  |  |
| Group | Within group number of adult males^1^ | 0.000 | 1.101 | 0.489 | 3.765 | 0.047 ; 2.146 | 1 | 0.052 |
|  | Between groups  number of adult males^1^ | NA | 4.766 | 1.358 | 5.726 | 1.997 ; 7.579 | 1 | **0.016** |
| Group | Food availability^1, 3^ | 0.234 | -1.078 | 0.596 | 2.544 | -2.319 ; 0.191 | 1 | 0.110 |
| Group | Observation hours^2, 4^ | 0.000 | 0.975 | 0.550 | 2.667 | -0.133 ; 2.125 | 1 | 0.102 |
| Residual |  | 12.36 |  |  |  |  |  |  |

Estimated variance components for the random effects and residuals come from the full model (model 1B). The column ‘Terms’ specifies whether the row refers to a random intercept or random slope component. Marginal effect sizes (R²), counting for the variance explained by fixed effects, was 0.68, while conditional R2, counting for the variance of both fixed and random effects, was 0.85; (1) test predictor; (2) control predictor; (3) z-transformed, mean and sd of the original values were 1.76 and 0.88, respectively; (4) z-transformed, mean and sd of original values were 2267.52 and 973.63, respectively, before being log-transformed; p-values in bold indicate a statistically significant effect (p < 0.05).

**Table S3: Determinants of yearly territory size for a model using the number of mature individuals (model 1C).**

| **Random effect** | **Terms** | **Variance** | **Estimate** | **SE** | **χ ²** | **95% CI** | **Df** | **P** |
| --- | --- | --- | --- | --- | --- | --- | --- | --- |
| Group | intercept | 8.587 | 4.851 | 3.757 |  | -2.368 ; 12.382 |  |  |
| Group | Within group number of mature individuals^1^ | 0.000 | - 0.310 | 0.180 | 2.129 | -0.675 ; 0.040 | 1 | 0.144 |
|  | Between groups  number of mature individuals^1^ | NA | 0.960 | 0.226 | 7.022 | 0.505 ; 1.390 | 1 | **0.008** |
| Group | Food availability^1, 3^ | 0.000 | - 0.985 | 0.552 | 2.829 | -2.319 ; 0.191 | 1 | 0.092 |
| Group | Observation hours^2, 4^ | 0.000 | 1.374 | 0.569 | 4.266 | -0.133 ; 2.125 | 1 | **0.038** |
| Residual |  | 13.11 |  |  |  |  |  |  |

Estimated variance components for the random effects and residuals come from the full model (model 1C). The column ‘Terms’ specifies whether the row refers to a random intercept or random slope component. Marginal effect sizes (R²), counting for the variance explained by fixed effects, was 0.67, while conditional R2, counting for the variance of both fixed and random effects, was 0.80; (1) test predictor; (2) control predictor; (3) z-transformed, mean and sd of the original values were 1.76 and 0.88, respectively; (4) z-transformed, mean and sd of original values were 2267.52 and 973.63, respectively, before being log-transformed; p-values in bold indicate a statistically significant effect (p < 0.05).

**Table S4: Determinants of monthly perceived neighbor pressure for a model using the number of adult males (model 2B).**

| **Random effect** | **Terms** | **Variance** | **Estimate** | **SE** | **χ ²** | **95% CI** | **Df** | **P** |
| --- | --- | --- | --- | --- | --- | --- | --- | --- |
| Group | intercept | 0.168 | -1.520 | 0.688 |  | -2.855 ; -0.153 |  |  |
| Group | Within group number of adult males^1^ | 0.000 | -0.082 | 0.089 | 0.688 | -0.256 ; 0.108 | 1 | 0.406 |
|  | Between groups  number of adult males^1^ | NA | -0.386 | 0.192 | 2.777 | -0.766 ; -0.017 | 1 | 0.095 |
| Group | Food availability^1, 3^ | 0.000 | 0.042 | 0.110 | 0.145 | -0.196 ; 0.271 | 1 | 0.703 |
| Group | Full tumescent swelling ratio^1, 4^ | 0.000 | -0.019 | 0.094 | 0.043 | -0.216 ; 0.164 | 1 | 0.835 |
| Group | Proportion of nulliparous females^1,5^ | 0.000 | -0.082 | 0.099 | 0.597 | -0.284; 0.127 | 1 | 0.439 |
| Group | Observation hours^2, 6^ | 0.071 | -0.001 | 0.169 | 0.001 | -0.333 ; 0.349 | 1 | 0.999 |
| Group | Sin (month)^2, 7^ | 0.000 | 0.438 | 0.124 | 7.679 | 0.197 ; 0.672 | 1 | **0.005** |
| Group | Cos (month)^2, 7^ | 0.000 | 0.252 | 0.160 | 2.170 | -0.052 ; 0.579 | 1 | 0.140 |
| Residual |  | 1.53 |  |  |  |  |  |  |

Estimated variance components for the random effects and residuals come from the full model (model 2B). The column ‘Terms’ specifies whether the row refers to a random intercept or random slope component. Marginal effect sizes (R²), counting for the variance explained by fixed effects, was 0.15, while conditional R2, counting for the variance of both fixed and random effects, was 0.26; (1) test predictor; (2) control predictor; (3) z-transformed, mean and sd of the original values were 1.79 and 1.35, respectively; (4) z-transformed, mean and sd of the original values were 0.085 and 0.070, respectively; (5) z-transformed, mean and sd of the original values were 0.081 and 0.061, respectively; (6) z-transformed, mean and sd of the original values were 241.52 and 82.33, respectively before being log-transformed; (7) transformed into a circular radiant variable; p-values in bold indicate a statistically significant effect (p < 0.05).

**Table S5: Determinants of monthly perceived neighbor pressure for a model using the number of mature individuals (model 2C).**

| **Random effect** | **Terms** | **Variance** | **Estimate** | **SE** | **χ ²** | **95% CI** | **Df** | **P** |
| --- | --- | --- | --- | --- | --- | --- | --- | --- |
| Group | intercept | 0.025 | -1.534 | 0.321 |  | -2.159 ; -0.915 |  |  |
| Group | Within group number of mature individuals^1^ | 0.000 | 0.052 | 0.038 | 1.626 | -0.027 ; 0.128 | 1 | 0.202 |
|  | Between groups  number of mature individuals^1^ | NA | -0.085 | 0.018 | 7.346 | -0.120 ; -0.048 | 1 | **0.006** |
| Group | Food availability^1, 3^ | 0.000 | 0.032 | 0.111 | 0.080 | -0.184 ; 0.262 | 1 | 0.776 |
| Group | Full tumescent swelling ratio^1, 4^ | 0.000 | 0.004 | 0.095 | 0.002 | -0.194 ; 0.175 | 1 | 0.962 |
| Group | Proportion of nulliparous females^1,5^ | 0.000 | -0.046 | 0.096 | 0.186 | -0.260; 0.158 | 1 | 0.665 |
| Group | Observation hours^2, 6^ | 0.079 | -0.017 | 0.175 | 0.008 | -0.368 ; 0.320 | 1 | 0.925 |
| Group | Sin (month)^2, 7^ | 0.000 | 0.442 | 0.123 | 7.871 | 0.195 ; 0.684 | 1 | **0.005** |
| Group | Cos (month)^2, 7^ | 0.000 | 0.255 | 0.160 | 2.181 | -0.064 ; 0.564 | 1 | 0.139 |
| Residual |  | 1.51 |  |  |  |  |  |  |

Estimated variance components for the random effects and residuals come from the full model (model 2C). The column ‘Terms’ specifies whether the row refers to a random intercept or random slope component. Marginal effect sizes (R²), counting for the variance explained by fixed effects, was 0.19, while conditional R2, counting for the variance of both fixed and random effects, was 0.25; (1) test predictor; (2) control predictor; (3) z-transformed, mean and sd of the original values were 1.79 and 1.35, respectively; (4) z-transformed, mean and sd of the original values were 0.085 and 0.070, respectively; (5) z-transformed, mean and sd of the original values were 0.081 and 0.061, respectively; (6) z-transformed, mean and sd of the original values were 241.52 and 82.33, respectively before being log-transformed; (7) transformed into a circular radiant variable; p-values in bold indicate a statistically significant effect (p < 0.05).

**Table S6: Determinants of monthly perceived neighbor pressure for a model using group size, which includes all months even in the absence of inter-group encounters.**

| **Random effect** | **Terms** | **Variance** | **Estimate** | **SE** | **χ ²** | **95% CI** | **Df** | **P** |
| --- | --- | --- | --- | --- | --- | --- | --- | --- |
| Group | intercept | 0.560 | -7.338 | 1.241 |  | -9.854; -4.856 |  |  |
| Group | Within group number of individuals^1^ | 0.056 | 0.339 | 0.148 | 3.315 | 0.031; 0.655 | 1 | 0.068 |
|  | Between groups  number of individuals^1^ | NA | -0.181 | 0.058 | 5.387 | -0.297; -0.067 | 1 | **0.020** |
| Group | Food availability^1,3^ | 0.000 | -0.220 | 0.321 | 0.457 | -0.836; 0.439 | 1 | 0.498 |
| Group | Full tumescent swelling ratio^1, 4^ | 0.000 | 0.537 | 0.255 | 2.478 | 0.054; 1.051 | 1 | 0.115 |
| Group | Proportion of nulliparous females^1,5^ | 0.814 | 0.435 | 0.547 | 0.545 | -0.630; 1.551 | 1 | 0.460 |
| Group | Observation hours^2, 6^ | 0.648 | 1.751 | 0.514 | 5.435 | 0.777; 2.699 | 1 | **0.019** |
| Group | Sin (month)^2,7^ | 0.223 | 0.986 | 0.438 | 3.863 | 0.121; 1.846 | 1 | **0.049** |
| Group | Cos (month)^2,7^ | 0.000 | 1.653 | 0.434 | 9.042 | 0.795; 2.536 | 1 | **0.002** |
| residual |  | 33.26 |  |  |  |  |  |  |

Estimated variance components for the random effects and residuals come from the full model. Full-null model comparison: LRT χ ² = 12.02, df = 5, P = 0.034. The column ‘Terms’ specifies whether the row refers to a random intercept or random slope component. Marginal effect sizes (R²), counting for the variance explained by fixed effects, was 0.19, while conditional R2, counting for the variance of both fixed and random effects, was 0.26; (1) test predictor; (2) control predictor; (3) z-transformed, mean and sd of the original values were 1.68 and 1.30, respectively; (4) z-transformed, mean and sd of the original values were 0.078 and 0.070, respectively; (5) z-transformed, mean and sd of the original values were 0.081 and 0.061, respectively; (6) z-transformed, mean and sd of the original values were 212.96 and 89.84, respectively before being log-transformed; (7) transformed into a circular radiant variable; p-values in bold indicate a statistically significant effect (p < 0.05). Sample size N = 556.

**Table S7: Determinants of monthly perceived neighbor pressure for a model using the number of adult males, which includes all months even in the absence of inter-group encounters.**

| **Random effect** | **Terms** | **Variance** | **Estimate** | **SE** | **χ ²** | **95% CI** | **Df** | **P** |
| --- | --- | --- | --- | --- | --- | --- | --- | --- |
| Group | intercept | 0.170 | -7.898 | 1.860 |  | -11.468; -4.200 |  |  |
| Group | Within group number of adult males^1^ | 1.645 | 0.601 | 0.733 | 0.653 | -0.866; 2.051 | 1 | 0.418 |
|  | Between groups  number of adult males^1^ | NA | -0.853 | 0.506 | 2.204 | -1.855; 0.166 | 1 | 0.137 |
| Group | Food availability^1, 3^ | 0.000 | -0.344 | 0.323 | 1.114 | -1.025; 0.294 | 1 | 0.291 |
| Group | Full tumescent swelling ratio^1, 4^ | 0.000 | 0.489 | 0.259 | 2.593 | -0.024; 1.005 | 1 | 0.107 |
| Group | Proportion of nulliparous females^1,5^ | 0.830 | 0.123 | 0.569 | 0.040 | -1.004; 1.299 | 1 | 0.841 |
| Group | Observation hours^2, 6^ | 0.243 | 1.629 | 0.385 | 6.729 | 0.840; 2.369 | 1 | **0.009** |
| Group | Sin (month)^2, 7^ | 0.194 | 1.018 | 0.432 | 4.109 | 0.121; 1.879 | 1 | **0.042** |
| Group | Cos (month)^2, 7^ | 0.000 | 1.828 | 0.438 | 9.727 | 0.930; 2.630 | 1 | 0.001 |
| residual |  | 34.15 |  |  |  |  |  |  |

Estimated variance components for the random effects and residuals come from the full model. Full-null model comparison: LRT χ ² = 6.54, df = 5, P = 0.25. The column ‘Terms’ specifies whether the row refers to a random intercept or random slope component. Marginal effect sizes (R²), counting for the variance explained by fixed effects, was 0.13, while conditional R2, counting for the variance of both fixed and random effects, was 0.23; (1) test predictor; (2) control predictor; (3) z-transformed, mean and sd of the original values were 1.68 and 1.30, respectively; (4) z-transformed, mean and sd of the original values were 0.078 and 0.070, respectively; (5) z-transformed, mean and sd of the original values were 0.081 and 0.061, respectively; (6) z-transformed, mean and sd of the original values were 212.96 and 89.84, respectively before being log-transformed; (7) transformed into a circular radiant variable; p-values in bold indicate a statistically significant effect (p < 0.05). Sample size N = 556.

**Table S8: Determinants of monthly perceived neighbor pressure for a model using the number of mature individuals, which includes all months even in the absence of inter-group encounters.**

| **Random effect** | **Terms** | **Variance** | **Estimate** | **SE** | **χ ²** | **95% CI** | **Df** | **P** |
| --- | --- | --- | --- | --- | --- | --- | --- | --- |
| Group | intercept | 0.650 | - 7.550 | 1.257 |  | -10.286; -4.966 |  |  |
| Group | Within group number of mature individuals^1^ | 0.018 | 0.250 | 0.121 | 3.058 | 0.002; 0.483 | 1 | 0.080 |
|  | Between groups  number of mature individuals^1^ | NA | - 0.219 | 0.074 | 5.044 | - 0.363; - 0.061 | 1 | **0.024** |
| Group | Food availability^1, 3^ | 0.000 | - 0.223 | 0.319 | 0.432 | - 0.859; 0.452 | 1 | 0.510 |
| Group | Full tumescent swelling ratio^1, 4^ | 0.015 | 0.494 | 0.269 | 1.925 | -0.019; 0.996 | 1 | 0.165 |
| Group | Proportion of nulliparous females^1,5^ | 0.734 | 0.388 | 0.525 | 0.528 | - 0.669; 1.532 | 1 | 0.467 |
| Group | Observation hours^2, 6^ | 0.595 | 1.751 | 0.500 | 5.503 | 0.719; 2.729 | 1 | **0.018** |
| Group | Sin (month)^2, 7^ | 0.160 | 1.004 | 0.421 | 4.173 | 0.164; 1.837 | 1 | **0.041** |
| Group | Cos (month)^2, 7^ | 0.000 | 1.713 | 0.438 | 8.950 | 0.814; 2.599 | 1 | 0.002 |
| residual |  | 34.32 |  |  |  |  |  |  |

Estimated variance components for the random effects and residuals come from the full model. Full-null model comparison: LRT χ ² = 10.80, df = 5, P = 0.055. The column ‘Terms’ specifies whether the row refers to a random intercept or random slope component. Marginal effect sizes (R²), counting for the variance explained by fixed effects, was 0.16, while conditional R2, counting for the variance of both fixed and random effects, was 0.21; (1) test predictor; (2) control predictor; (3) z-transformed, mean and sd of the original values were 1.68 and 1.30, respectively; (4) z-transformed, mean and sd of the original values were 0.078 and 0.070, respectively; (5) z-transformed, mean and sd of the original values were 0.081 and 0.061, respectively; (6) z-transformed, mean and sd of the original values were 212.96 and 89.84, respectively before being log-transformed; (7) transformed into a circular radiant variable; p-values in bold indicate a statistically significant effect (p < 0.05). Sample size N = 556.

**Fig.S1. Map illustrating the variation in yearly territory sizes for the four studied groups. Minimum and maximum territory sizes are depicted by the minimum convex polygons (MCP) calculated from the 95% fixed kernels of all locations. Numbers refer to the year, letters refer to the group: N North, M Middle, S South and E East group.**

**
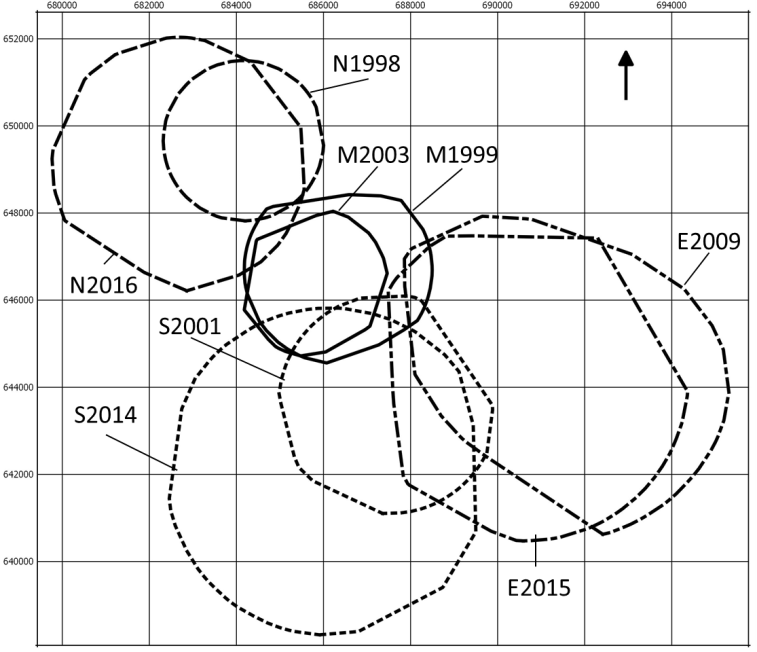
**
